# Distribution of hygiene kits during a cholera outbreak in Kasaï-Oriental, Democratic Republic of Congo: a process evaluation

**DOI:** 10.1186/s13031-020-00294-w

**Published:** 2020-07-24

**Authors:** Lauren D’Mello-Guyett, Katie Greenland, Sharla Bonneville, Rob D’hondt, Maria Mashako, Alexandre Gorski, Dorien Verheyen, Rafael Van den Bergh, Peter Maes, Francesco Checchi, Oliver Cumming

**Affiliations:** 1 Faculty of Infectious and Tropical Diseases, London School of Hygiene and Tropical Medicine, London, UK; 2 Environmental Health Unit, Médecins Sans Frontières, Brussels, Belgium; 3 Médecins Sans Frontières, Kinshasa, Democratic Republic of Congo; 4 LuxOR, Luxembourg Operational Research Unit, Médecins Sans Frontières, Luxembourg City, Luxembourg; 5 Faculty of Epidemiology and Population Health, London School of Hygiene and Tropical Medicine, London, UK

**Keywords:** Cholera, Outbreaks, Emergency, Water, Sanitation, Hygiene, Process evaluation

## Abstract

**Background:**

Cholera remains a leading cause of infectious disease outbreaks globally, and a major public health threat in complex emergencies. Hygiene kits distributed to cholera case-households have previously shown an effect in reducing cholera incidence and are recommended by Médecins Sans Frontières (MSF) for distribution to admitted patients and accompanying household members upon admission to health care facilities (HCFs).

**Methods:**

This process evaluation documented the implementation, participant response and context of hygiene kit distribution by MSF during a 2018 cholera outbreak in Kasaï-Oriental, Democratic Republic of Congo (DRC). The study population comprised key informant interviews with seven MSF staff, 17 staff from other organisations and a random sample of 27 hygiene kit recipients. Structured observations were conducted of hygiene kit demonstrations and health promotion, and programme reports were analysed to triangulate data.

**Results and conclusions:**

Between Week (W) 28–48 of the 2018 cholera outbreak in Kasaï-Oriental, there were 667 suspected cholera cases with a 5% case fatality rate (CFR). Across seven HCFs supported by MSF, 196 patients were admitted with suspected cholera between W43-W47 and hygiene kit were provided to patients upon admission and health promotion at the HCF was conducted to accompanying household contacts 5–6 times per day. Distribution of hygiene kits was limited and only 52% of admitted suspected cholera cases received a hygiene kit. The delay of the overall response, delayed supply and insufficient quantities of hygiene kits available limited the coverage and utility of the hygiene kits, and may have diminished the effectiveness of the intervention. The integration of a WASH intervention for cholera control at the point of patient admission is a growing trend and promising intervention for case-targeted cholera responses. However, the barriers identified in this study warrant consideration in subsequent cholera responses and further research is required to identify ways to improve implementation and delivery of this intervention.

## Introduction

Cholera is a diarrhoeal disease transmitted through faecal-oral routes and caused by the pathogenic bacteria *Vibrio cholerae* O1 and O139. It remains a leading cause of infectious disease outbreaks globally [1, 2], and a major public health threat in complex emergencies [3, 4]. The Democratic Republic of Congo (DRC) contributes an estimated 189,000 (5–14%) of the annual estimated 1.3–4.0 million cholera cases worldwide [4] and is considered a hotspot for cholera transmission regionally [5–7]. Cholera has been endemic in DRC since 1978 [8], and repeated complex emergencies have contributed to regular outbreaks [8–10]. In 2018 alone, 28,332 cholera cases and 890 deaths were recorded [11].

Spatiotemporal analyses suggest that transmission is localised to the households of cholera cases and household contacts of cases have up to a 100-fold greater risk of infection than those outside of the household [12–14], with risk greatest during the first 7 days after onset of a case’s symptoms [15–17]. Evidence demonstrates that within-household transmission (i.e. human-to-human transmission) of cholera occurs through shared drinking water [18], contaminated food [19] and caring for the ill, due to prolific shedding from symptomatic and asymptomatic cases which can continue up to 14 days after onset of symptoms [20]. Models also show that within-household transmission contributes more to the explosive nature of epidemics than transmission through in the community such as environment-to-human transmission from contaminated water sources [12, 21–23]. Household-level water, sanitation and hygiene (WASH) interventions targeting within-household may thus be important in combatting cholera outbreaks [24–26], and can align with case-centred strategies for effective disease control [27–29].

“Hygiene kits” are a household-level WASH intervention recommended for use during cholera outbreak responses and in other crises contexts [30–33]. Selection of hygiene kit contents differs between organisations but they typically include a jerrycan (e.g. 10 to 20 litres (L)) for water collection and storage, soap, point of use (POU) water treatment product/s (e.g. chlorine, filters and/or flocculant disinfectants) and a handwashing device (e.g. a 10-L bucket with tap). Some guidelines specify that hygiene kits should contain components in sufficient quantities for one month’s use by an “average sized” household [31, 32], whereas others recommend the inclusion of other components (e.g. toothbrushes, menstrual hygiene management materials) appropriate for populations affected by other types of crises [33, 34]. Distribution of a hygiene kit to a cholera cases when they are admitted to a Cholera Treatment Centre (CTC) or Cholera Treatment Unit (CTU) has been recommended in the Médecins Sans Frontières (MSF) guidelines “*Management of a Cholera Epidemic*” since 2017 [30]. This is based on previous research which found that the distribution of hygiene kits, or their component parts [24, 35], were effective in reducing cholera transmission in Bangladesh [25] and Haiti [36], and the burden of other diarrhoeal diseases [37–39]. However, hygiene kit distribution in outbreak response has not been widely published and is not common in cholera outbreaks [24, 40–42], due in part to a lack of evidence on effectiveness [24, 43], transferability and scalability across contexts [40].

Hygiene kit distribution, like many public health interventions, is a complex intervention featuring several interacting components, and their effectiveness may vary across populations, settings and delivery modalities [44–46]. Process evaluations of complex interventions are increasingly conducted to help explain observed outcomes in intervention studies [47–51] and envision whether the intervention will achieve its intended effects in other contexts or scales [51, 52]. The process evaluation framework also allows implementation and change processes to be explored [47], the utility of theories underpinning intervention design such as hygiene kit distribution from health care facilities (HCFs) to be examined [53] and questions or hypotheses for future research to be generated. To date there have been no published process evaluations of the deployment of hygiene kits in cholera outbreaks.

We adapted conventional process evaluation methods developed for use in health impact trials to evaluate the distribution of hygiene kits by MSF during a cholera outbreak response in Kasansa district, Kasaï-Oriental province, DRC. This process evaluation ran in parallel with a prospective cohort study to assess the effect of the intervention on cholera incidence among household contacts of admitted cholera cases which will be published at a later date. This process evaluation sought to identify the successes and barriers of the hygiene kit distribution strategy for cholera control in order to understand delivery, use and scalability, and to propose recommendations to optimise future programmes. Three evaluation domains were explored including the implementation of the intervention, participants’ responses to the intervention and the context in which it was delivered.

## Methods

### Epidemiology of cholera in Kasansa, Kasaï-oriental

The DRC Programme National d’Elimination du Choléra et de Lutte contre les autres Maladies Diarrhéiques (PNECHOL-MD), or National Program for the Elimination of Cholera and other Diarrhoeal Diseases, issued a country-wide alert of one laboratory confirmed cholera case in Kasansa district, Kasaï-Oriental province, DRC, on 9th August 2018 (Epidemiological Week 28 (W28)) [54, 55]. A second alert and call for assistance came from the PNECHOL-MD in W34 [56–59].

Between W28–42, there were 443 suspected cholera cases and 29 deaths across Kasansa. MSF joined in W43, 16 weeks after the first laboratory-confirmed case, for 5 weeks between 22nd October to 23rd November 2018 (W43–47). A further 224 suspected cholera cases and 3 deaths occurred between W43–47 [55, 56, 58–66]. There was a high overall case fatality ratio (CFR) of 5% and Attack Rate (AR) of 0.28% between W28–47 [66].

### Study setting and timeline of response

In 2018, there were an estimated 230,000 people living in Kasansa across 18 communities (Aires de Santé) [54]. Kasansa is a relatively homogeneous district in terms of socioeconomic composition of the population and agriculture-based income, and the local government had limited resources for health care [59, 65]. A high burden of cholera with high CFR had been observed throughout 2017 and 2018 across Kasaï-Oriental [9, 11, 67], and MSF had responded to other outbreaks earlier in 2018 [68]. Aside from MSF, there were few other public health programmes operating in Kasansa. Other non-governmental organisations (NGOs) and government programmes included hygiene education, malnutrition awareness and malaria prevention.

**Fig. 1:**
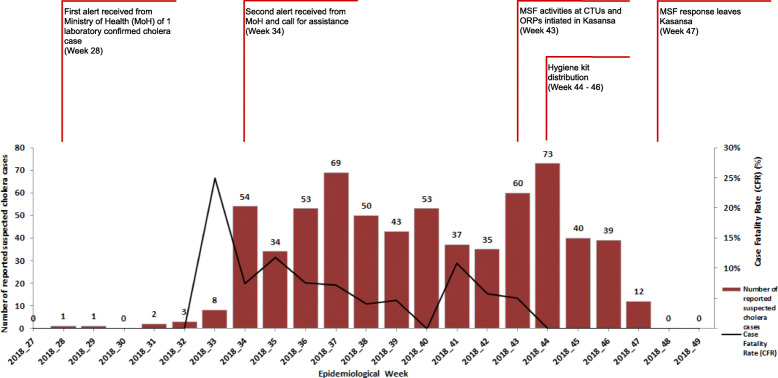
Epidemiology of a cholera outbreak in Kasansa, Kasaï-Oriental, Democratic Republic of Congo (DRC) and timeline of response

The cholera response in Kasansa was led by the Ministry of Health (MoH). MSF supported seven government HCFs, two CTUs and five Oral Rehydration Points (ORPs) to provide case management, essential medicine supply, enhanced surveillance, community-level health promotion, and infrastructure improvements. Due to a high CFR and low attendance at HCFs by cases [54, 56, 66], outreach community health workers (CHWs) and an ambulance were deployed from W43. A total of 196 suspected cholera cases (75% of total reported suspected cases) were admitted across all seven MSF-supported HCFs (121 in CTUs and 75 in ORPs) between W43–47. Hygiene kits were distributed with health promotion messaging to cholera patients admitted to the two MSF-supported CTUs, but not to patients at the ORPs, from W44–46 (Fig. 1).

### Theory of change

**Fig. 2:**
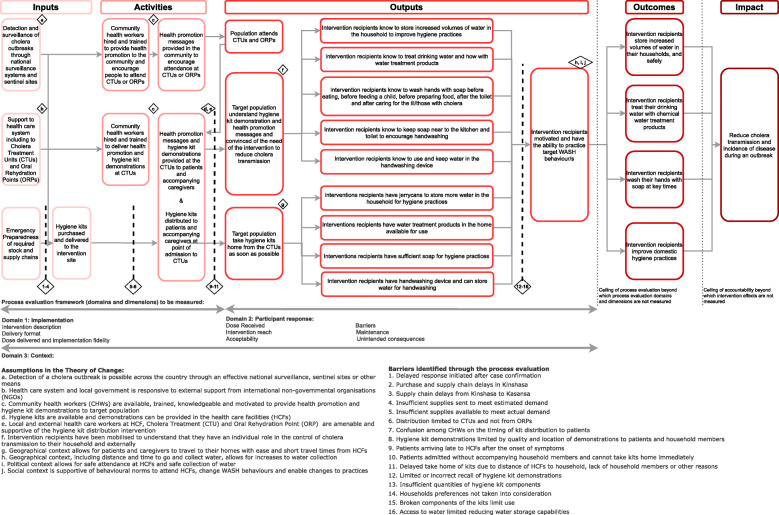
A Theory of Change (ToC), including related assumptions, process evaluation framework and identified barriers from the analysis, for the distribution of hygiene kits and health promotion to cholera cases and their households

Hygiene kit distribution was one component of the overall cholera response and a Theory of Change (ToC) was developed to provide a framework for the study (Fig. 2). Figure 2 shows how the effectiveness of the hygiene kit to reduce transmission of cholera among household contacts of cases and overall cholera incidence (Impact) may be influenced by factors along the ToC, beginning with i) national and local emergency preparedness supplies and the supply and delivery of hygiene kits to the intervention site (Inputs); which in turn determines ii) adequate health promotion, hygiene kit demonstrations in the CTUs and timely distribution of the kits to the target population at the point of admission (Activities); which leads to iii) the target population understanding the health promotion and hygiene kit demonstrations delivered at the CTUs and intending to take the kits home as soon as possible (Outputs); and finally, iv) intervention recipients who are motivated and have the ability to practice the target WASH behaviour/s (Outputs & Outcomes). Other factors and assumptions that needed to hold true for change to occur as predicted are also illustrated in the ToC.

### Process evaluation framework

A process evaluation framework, including the domains, dimensions, research questions and data collection methods (Table 1), was developed using the process evaluation literature [47, 49, 52, 69], as well as relevant published applications in public health [70, 71] together with complex system theory [53]. Accordingly, this mixed methods study included quantitative measures of intervention activities and qualitative exploration of the interaction between the three domains of intervention implementation, participant response to the intervention, and the context that mediated this relationship. The domain of intervention context was populated according to seven established “pillars” (geographical, political, ethical, legal, epidemiological, socio-cultural, and socio-economic structures) and at the macro (i.e. national systems and structures), meso (i.e. institutional and community) and micro (i.e. participants and local surroundings) levels, according to models developed in the literature [49, 50, 72].

**Table 1:** Process evaluation framework: domains, dimensions, study population, data sources and data types

Process evaluation domains and dimensions	Research question	Core information sought	Study population	Data source	Data type
**Domain 1: Implementation**					
1. Intervention description	a. What was the design of the intervention?	Overall design of the intervention including site, population, health care facility and structure, rationale and timeline	MSF staff implementing the intervention (e.g. field coordinators, medical, logistics, WASH, supply and health promotion coordinators)	Semi-structured interviews (SSIs) and intervention reports and surveillance data from the local & national government including georeferenced maps	Qualitative
	b. What are the components of the intervention?	Content of the intervention including the intervention components and selection		MSF cholera guidelines, supply and kit catalogues, intervention reports and activity records (e.g. supply chain freight manifests, purchase orders, distribution lists, attendance registers)	Qualitative
2. Delivery format	Where and when was the intervention delivered?	Description of targeted area, population size, health care facility structure and environment, and timeline of the intervention	MSF staff, local government and national agencies (e.g. WASH and health clusters, PNECHOL-MD)	SSIs, intervention timeline and intervention reports	Qualitative/ Quantitative
			MSF staff, local government and other organisations (e.g. Save The Children, UNICEF, Solidarities International, Catholic Relief Services, Action Aid)	SSIs, intervention timeline and intervention reports	Qualitative/ Quantitative
	What other interventions (WASH and non-WASH) were provided by MSF?	Documentation of other interventions locally and nationally including the number of “competing” programmes, or components reaching the target population	MSF staff, local government and other organisations	SSIs and intervention reports	Qualitative/ Quantitative
	What other agencies were involved in implementation?	Documentation of the other agencies and government structures operating in the targeted area, including their roles and perceptions on the intervention	MSF staff, local government and other organisations	SSIs and intervention reports	Qualitative
	How was the intervention demonstrated and explained to users?	Documentation of the delivery format, timing and interaction with intervention recipients	MSF staff (e.g. CHWs)	Structured observations	Qualitative/ Quantitative
			MSF staff & intervention recipients	SSIs and intervention reports	Qualitative
	What resources were used to implement the intervention?	Human, material and financial resources utilised by the intervention	MSF staff, local government and other organisations	SSIs, intervention reports, activity records and budgets	Qualitative/ Quantitative
3. Dose delivered and implementation fidelity	How many interventions were delivered?	Number of interventions delivered and number of planned interventions		SSIs, intervention reports and activity records and surveillance data	Quantitative
	Was the intervention delivered as planned?	Documentation of the content, quality, successes and challenges of the intervention delivered	MSF staff & intervention recipients	SSIs and intervention reports	Qualitative/ Quantitative
**Domain 2: Participant response**					
4. Dose received	How many interventions were received?	Number of interventions received in the households of recipients	Intervention recipients	SSIs	Qualitative
5. Intervention reach	How many people interacted with the intervention? And their uptake of the intervention?	Number of people in the household who interacted with the intervention and use	Intervention recipients	SSIs	Qualitative
6. Acceptability	What were the levels of participation and satisfaction?	Comprehension of emotional responses to the intervention, acceptability of the intervention and component preferences	Intervention recipients	SSIs	Qualitative
7. Barriers	What were the barriers to using the intervention?	Obstruction (physical and/or emotional barriers) to the intervention and concerns with the intervention	Intervention recipients, MSF staff, local government and other organisations	SSIs	Qualitative
8. Maintenance	How and why was the intervention sustained over time (or not)?	Retention of key messages, target behaviours and reflections of the intervention	Intervention recipients	SSIs	Qualitative/ Quantitative
9. Unintended consequences	What effects were not captured or were there unexpected outcomes, both related to the intervention and unrelated care?	Reasons for any deviation from the intended activities, interaction with and use of the intervention	Intervention recipients, MSF staff, local government and other organisations	SSIs	Qualitative
**Domain 3: Context**					
Context	What was the context?	Characteristics of the delivery context (geographical, political, legal, ethical, epidemiological, sociocultural, socioeconomic)	MSF staff, local government and other organisations	SSIs, intervention reports, activity records and budgets	Qualitative
	What external factors affected the implementation and the outcome?	Organisational context: culture, agenda, priorities, leadership styles and perceptions of leaders, perceptions on research and evaluation, and other contextual factors	MSF staff, local government and other organisations	SSIs and intervention reports	Qualitative

*CHWs* Community Health Workers, *MSF* Médecins Sans Frontières, *PNECHOL-MD* Programme National d’Elimination du Choléra et de Lutte contre les autres Maladies Diarrhéiques, *SSIs* Semi-structured interviews, *WASH* Water, sanitation and hygiene

### Data collection

Most data collection was prospective, pre-specified and collected during and immediately following the MSF response, between October–December 2018 (W43–52). Some data, including intervention reports and additional surveillance data, were collected *post-hoc* between December 2018–February 2019 (W52–9).

The evaluation team comprised five experienced Congolese enumerators, all of whom held Bachelor’s degrees and were MSF staff, partnered with five local less-experienced Congolese enumerators from Kasaï-Oriental, who had up to secondary level education and were hired on temporary contracts for the study period. All data collection was conducted with assistance from two female international investigators (one British and one Canadian), both of whom had Master’s degrees. Prior to data collection, a five-day training was led by the two international investigators in Kinshasa to introduce the study, methods, ethics of research and to pilot all data collection tools. A two-day training was provided to the local Congolese enumerators at the study site. The evaluation team were MSF staff and study participants knew of the researchers’ affiliations to the organisation. The evaluation team was not involved in the design or implementation of the intervention.

All tools were written in English, translated to French, piloted and translated to the local language, Tshiluba. The study is reported in accordance with the COREQ checklist for qualitative studies [73]. Specific data collection methods are described below and summarised in Table 1.

### Semi-structured interviews and observations

Five Congolese enumerators conducted semi-structured interviews (SSIs) between W45–47 at households of a simple random sample of households enrolled in the parallel prospective cohort study who received a hygiene kit at an MSF-supported CTU. These SSIs lasted approximately 30 min and followed a topic guide including reported and observed measures of hygiene kit use to explore the participant response domain. SSIs with households were conducted until perceived data saturation.

SSIs with a purposive sample of MSF and non-MSF “implementers” were also conducted by three enumerators (one Congolese, one Canadian and one British) between W43–48, also until saturation. Implementers conducting activities in the study site were informed of the study in advance and requested to participate in SSIs. SSIs followed another topic guide to explore the implementation and context domains. Interviews were conducted for 30–45 min in participants’ offices or at the CTUs.

### Structured observations

Two Congolese enumerators conducted weekly unannounced visits to the two CTUs to observe hygiene kit demonstrations and health promotion sessions. A structured form was used to record details about the implementation and participant response domains.

### Intervention reports, activity records and budget

A total of 34 intervention documents were collected from implementers, local government and other organisations. Details of the strategy and description of the interventions were checked against guidelines [30], equipment catalogues [74] and internal policy documents. Attendance at hygiene kit demonstrations and health promotion sessions, and hygiene kit distribution lists were all recorded. Surveillance data were collected from the local and national government to describe the epidemiological context of the intervention. Any details of implementation and context domains were extracted from these reports.

### Data management and analysis

SSI data were collected on tablets through the KOBO Toolbox platform (Harvard Humanitarian Initiative, Cambridge, MA, USA), which allows a combination of quantitative questions and audio recordings of the interviews. Field notes were also taken throughout the interview by the enumerators. Transcriptions from the audio recordings and field notes were made in MS Word (Microsoft, Redmond, VA, USA). Data from structured observations were collected on paper forms and transcribed to MS Excel (Microsoft, Redmond, VA, USA).

Quantitative data from surveillance, intervention documents and structured observations were entered into MS Excel to form a single dataset for analysis. Data on the implementation and receipt were cleaned and analysed in Stata 15 (StataCorp, College Station, TX, USA).

Qualitative analysis from the SSI transcriptions, structured observations and intervention documents was conducted in NVivo 11 (QSR International, Doncaster, Victoria, Australia) and analysis was based on thematic content analysis [75, 76] and example papers [69]. Following an iterative process to analyse the data, data were coded deductively according to the pre-specified domains and dimensions of our process evaluation framework.

## Results

### Description of study participants

Household SSIs featured 27 respondents (13 female; average age 43 years). All respondents were married with four children on average, and up to 22 people lived in their households. No household refused to participate in the study. All respondents were engaged in agriculture and/or artisanal diamond mining. None of the respondents had themselves been admitted to a CTU or ORP and all were relatives of the admitted case.

SSIs were conducted with 17 implementers (seven MSF, four local government and six from NGOs), three of whom were female. No implementer or organisation refused to participate. Implementers from MSF and NGOs had on average 3 years of experience in cholera outbreaks, and over 5 years working with NGOs. Government respondents had less than a year working in cholera outbreaks, and over 5 years of experience working in government.

### Process evaluation findings

Following indexing of findings, a narrative was synthesised for each domain and dimension of the process evaluation. Table 2 presents illustrative quotations and are cited in the text. Barriers to intervention implementation and participant response are indicated below by numbers in square brackets e.g. **[Barrier 1]** and mapped back onto the ToC (Fig. 2).

**Table 2:** Illustrative quotations from hygiene kit recipients and programme implementers from a cholera outbreak in Kasansa, Kasai-Oriental, Democratic Republic of Congo

*Process evaluation dimension*	*Quotation Number*	*Quotation*
Delivery format	1	*“The cholera programmes are challenging from logistics point of view. In Mbuyi Mayi* [provincial capital]*, it’s not possible to find P&G Purifier of Water™ and Aquatabs™. So, everything comes from Goma or Kinshasa. All of our staff come from Goma or Kinshasa. And our money does too- we are waiting for people to make signatures on the delivery of products and money to pay local RECOs* [community health workers]*. “* - Respondent #12, female
	2	*“It was loud in the CTU, and new patients were always arriving. Because it was small, there was not a big space for the demonstrations. The RECO* [community health workers] *also had to repeat parts many times. Sometimes there were differences between sessions.”* - Respondent #2, male
Dose delivered and implementation fidelity	3	*“At the beginning, I gave the kit to the cases who had confirmed cholera. Then I gave them to all the patients. But some patients had no family. I had to give directly to the patient.”* - Respondent #3, female
	4	*“In the intervention, we gave the kits at admission. But this was not happening at the beginning. At the beginning, I gave the kit to the cases who had confirmed cholera …. At the end I was giving them to everyone at admission to the CTU.”* - Respondent #3, female
Dose received, reach and acceptability	5	*“Our water source is far away and has a lot of sediment, maybe 45 min, I walk. And the filter valve provided to filter the water does not easily pass water especially when the water is dirty from the river. It takes a long time to filter.”* - Respondent #21, female
	6	*“If I have the necessary means and enough, I will buy kits for my wives, but the lack of money makes it difficult. I have 3 wives in three separate houses and there are not buckets to share.”* - Respondent #24, male
Barriers to intervention use	7	*“Yes, I need several more elements than just the hygiene kit. I would like some foufou (maize flour), milk, clothes for the family, land my children need money to support school.”* - Respondent #21, male
	8	*“So, we have no measure of impact for the kits we planned gave out. We need to conduct post distribution monitoring to see what has been used in kits and to also check precisely on the diversity of use. Some of the utensils of the kit including the bucket with tap served as storage of things rather than handwashing bucket. We also need to check if supplies need to be redistributed.”* - Respondent #5, male
	9	*“So, I have the feeling that all of us are doing really short interventions, like the distribution of Aquatabs™, P&G Purifier of Water™, chlorination points. But the biggest challenge here is the lack of water. So, we were discussing also with the Hub, the WASH Cluster in Kasaï-Oriental today and also, he was thinking that maybe would have been better to focus funding durable solutions. So yes, it’s true that there is the need now but maybe now that the cholera cases are reducing, we would be better looking at...maybe...financing new water points or chlorination points for 2 months. We could decide to rehabilitate the existing infrastructure or having, in this case, huge funding for rehabilitating the water gravity scheme that is here.”* - Respondent #12, male

### Domain 1: implementation

#### Intervention description

Each cholera case and their accompanying household received a hygiene kit containing a 1 kg bar soap; 60 sachets of flocculant disinfectant (P&G Purifier of Water™, Procter & Gamble and Centers for Disease Control and Prevention, Pakistan) or 120 chlorine tablets (Aquatabs™, Medentech, Wexford, Ireland) estimated to be sufficient to treat 20-L of water per day for 30 days; a handwashing device of a 10-L bucket with tap and lid; and, a 20-L jerrycan. The appropriate water treatment product provided was determined based on the water source reportedly used by the household (flocculant-disinfectant for generally turbid open surface water sources; chlorine disinfectant for protected sources). Hygiene kits were distributed by local, MSF-trained CHWs to patients and their accompanying household members at the CTUs, with hygiene kit demonstrations provided 5–6 times per day.

#### Delivery format

The response was initiated midway through the cholera outbreak **[Barrier 1]** and quantitative findings indicated that hygiene kits distribution was limited to W44–46 **[Barrier 2].** Access and delivery of interventions to remote populations came with steep financial costs and required an influx of non-local staff and supplies, as mentioned in qualitative interviews with implementers (Quotation 1) **[Barrier 3 & 4]**.

Most hygiene kits were distributed on the day of admission (71%), with the remainder 1–3 days after **[Barrier 7]**. Between W44–46, 131 hygiene kit demonstrations and health promotion sessions were attended by 749 people at the two CTUs. Structured observations suggested good adherence of the demonstrations to hygiene kit contents, described in Table 3, and that these were well received by the population and involved participants attending the CTUs. However, demonstrations were often interrupted by noise and other distractions (Quotation 2) **[Barrier 8]**.

**Table 3:** Content and delivery of health promotion and hygiene kit demonstrations at two Cholera Treatment Units (CTUs)

Observation site	Content of health promotion and hygiene kit demonstration														Comments on the delivery of session, approach and activities
	Hygiene kit component demonstrated					Health promotion messages included								Total components of a session	
	*Jerrycan (20 l (L))*	*P&G Purifier of Water™ (flocculant disinfectant)*	*Aquatabs™ (chlorine tablets)*	*Soap (1 kg bar soap)*	*Handwashing device (10-L bucket with tap)*	*Cholera transmission* (e.g. *F-diagram*)	*Encouraging care-seeking behaviour to HCFs*	*Treatment at MSF facilities is free*	*Increase water stored in the household (by using the jerrycan)*	*Boil or treat drinking water*	*Limit open defecation*	*Practice safe corpse preparation*	*Wash hands at key times (before eating, before food preparation, after toilet, after changing a baby’s nappy, after caring for the ill/contact with a cholera case)*		
*Nsenga Nsenga CTU*	✓	✓	×	✓	×	✓	×	×	✓	✓	×	×	✓	**6**	Demonstrations were conducted with a picture board but were often didactic and attendees were not able to ask questions or demonstrate recall of the messages or demonstration
*Nsenga Nsenga CTU*	✓	✓	✓	✓	✓	✓	×	×	×	✓	×	×	✓	**8**	CHWs instigated a question and answer game to check respondents understanding.
*Lukalaba CTU*	✓	✓	✓	✓	✓	✓	✓	✓	×	✓	×	×	✓	**10**	Demonstration of the hygiene kit was conducted through a picture board; CHWs responded to questions and encouraged use of the kits by households as soon as possible
*Lukalaba CTU*	✓	×	✓	✓	×	✓	✓	✓	✓	✓	✓	✓	✓	**11**	CHWs repeatedly explained that the kit is only given to those with cholera patients in the house; and that households should start using them immediately
*Lukalaba CTU*	✓	✓	✓	✓	✓	✓	×	×	×	✓	×	×	✓	**8**	CHWs paused to take questions, ask the attendees to repeat the demonstrations and pauses to check any responses; CHWs emphasised the use of the kit by all household members.
*Frequency among sessions*	**5**	**4**	**4**	**5**	**3**	**5**	**2**	**2**	**2**	**5**	**1**	**1**	**5**	*–*	*–*

*CHWs* Community Health Workers, *CTU* Cholera Treatment Unit, *HCF* Healthcare Facility, *MSF* Médecins Sand Frontières

#### Dose delivered and implementation fidelity

**Fig. 3:**
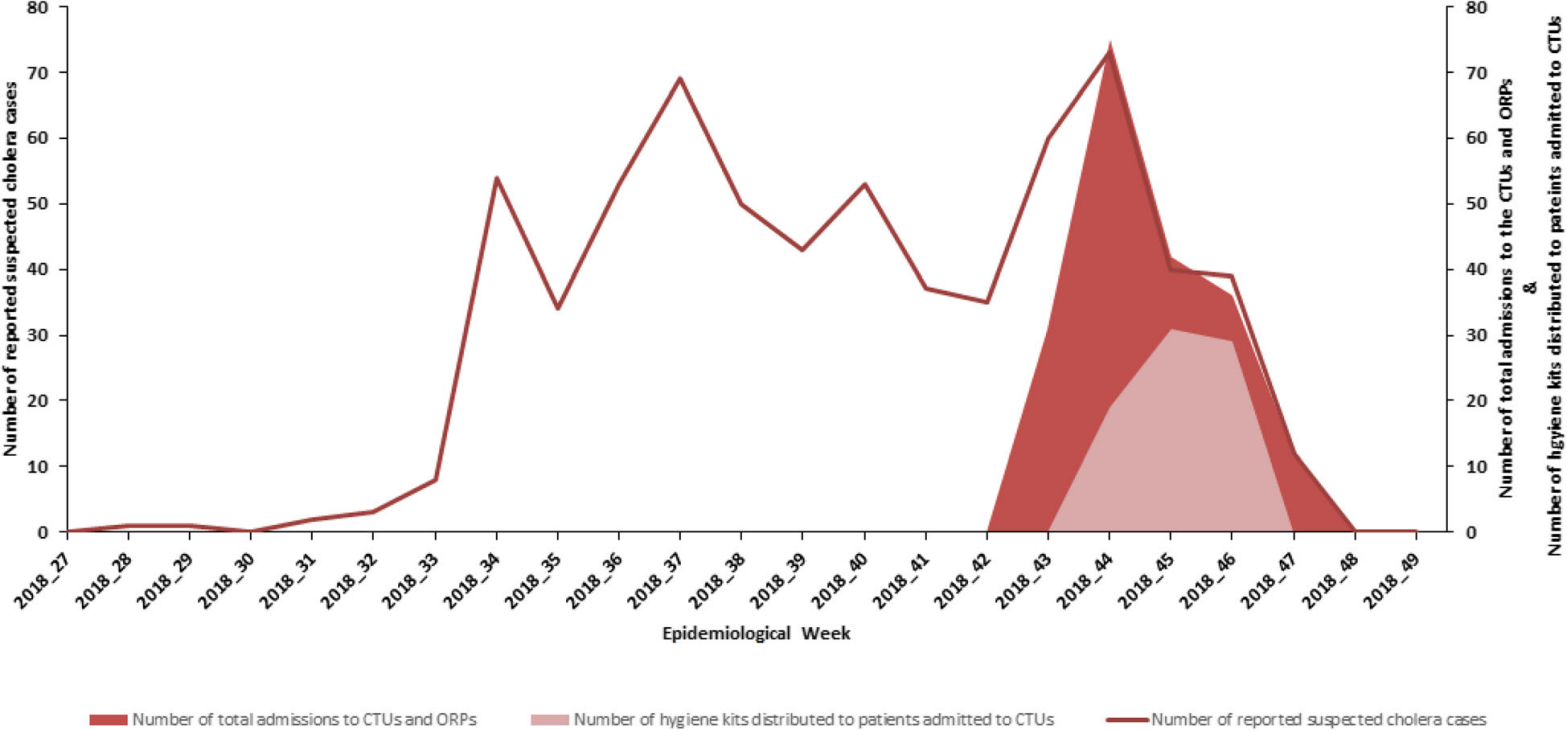
Coverage of hygiene kits distributed to patients between Week 43–462,018 of a cholera outbreak in Kasansa, Kasaï-Oriental, DRC

MSF estimated 250 kits would be required for the response, but only 165 kits were delivered to Kasansa. This was insufficient for the 196 patients admitted between W43–47 **[Barriers 4 & 5]**. Moreover, quantitative findings indicated only 79 admissions, or their accompanying household members, received a hygiene kit, namely 52% of the 153 admissions across all seven HCFs during the period when hygiene kits were available between W44–46 or 40% of the 196 suspected cases admitted to MSF-supported facilities between W43–47 (Fig. 3). The 86 unused kits were donated to local government when MSF left Kasansa.

Reasons given by implementers for the low coverage included the late arrival of the kits to the project site **[Barriers 1–3]**; CHWs only distributing kits from the two CTUs and not the five ORPs **[Barrier 6]**; patients without accompanying household members which caused confusion as to whom the kit should be given **[Barrier 10**]; and, incorrect timing of distribution (i.e. giving kits at exit rather than at admission) which had to be re-emphasised multiple times to CHWs (Quotation 3 & 4) **[Barrier 7]**.

### Domain 2: participant response

#### Dose received, reach and acceptability

In interviews with intervention recipients, most regarded the intervention to be useful in their households, with preference for the soap and the handwashing device. It was reported by interviewees that they had not shared or sold any components. Distance to HCFs was self-reported as a barrier to seeking care by households, and the weight and size of the kit was cited as an issue when taking the kit home (Quotation 5) **[Barrier 10]**. Yet results indicate that, on average, patients arrived at the HCFs and were admitted within 1 day of the onset of symptoms (median 1 day, range 0–10 days) **[Barrier 9]**, and all interviewed households reportedly brought the hygiene kit directly home within 3 days of kit receipt and within 7 days after the onset of symptoms (median 3 days, range 1–6 days) **[Barrier 11]**.

All interviewed households attended a hygiene kit demonstration, and respondents reported that they understood how the kits should be used. This translated to self-reported changes in the targeted WASH behaviours among these households. Interviewed recipients reported using all components of the hygiene kit at varying frequencies. The handwashing device and jerry can were reportedly used two-to-three times per day whereas POU water treatment was used between once a day to three times a week. Recall of when and how to treat drinking water was frequently incorrect **[Barrier 12]** and self-reported adherence to POU water treatment was low.

The handwashing device and jerrycan were observed to be in use during household SSIs (i.e. water available in either container), and soap was both observed to be in use (i.e. visible bubbles, or visibly smaller in size) and located next to the handwashing device or cooking area. These practices mirrored the observed emphasis that CHWs placed on handwashing and use of soap in the hygiene kit demonstrations and health promotion provided at the CTUs (Table 3).

#### Barriers to hygiene kit use

An inadequate quantity of soap was the most cited barrier to using the hygiene kit, particularly among larger families as all households received the same quantity of soap irrespective of household size **[Barrier 13]**. Several interviewees from polygamous households reported that kits were either not shared among co-wives and respective dwellings or, if shared, that quantities were insufficient (Quotation 6)*.* Similarly, one 20-L jerrycan for larger households was insufficient for their water storage needs, and households would have preferred a larger vessel (reported range 30–60-L). Other preferred items included money, more soap, food and clothes (Quotation 7) **[Barrier 14]**.

Implementers from MSF, local government and other NGOs also felt that quantities were insufficient for average-sized households **[Barrier 13]**, and repeated delivery would be required to facilitate effective disease control (Quotation 8). Many also felt that since the hygiene kit generally reduced risk of diarrhoeal diseases, households should be provided with enough materials to maintain use for longer than the outbreak. Implementers were also concerned that raising awareness of cholera and distribution of hygiene kits were limited actions, particularly when a population has only basic or limited access to water supply (e.g. river and surface water sources) (Quotation 9) **[Barrier 16]**.

#### Maintenance

Sustained use of the hygiene kits was difficult among interviewed households. Most households reported that they were unable to continue using the kit beyond 2 to 3 weeks, rather than the intended 1 month **[Barrier 13]**. Some parts of the kit were broken such as the tap on the handwashing device **[Barrier 15]***.* Enthusiasm to continue using the kit was high, although the availability of stored water and water inside the handwashing device was affected by distances and time to water supply. All households reported over 5 km distances to water sources **[Barrier 16]**.

#### Unintended consequences

There were no unintended consequences of hygiene kit use reported among intervention recipients. However, among the general population there was tension between households who had attended the CTU and received a hygiene kit and households who had attended the ORPs and not received a hygiene kit. Additionally, there was no retroactive distribution of kits to patients admitted to CTUs prior to the arrival of the hygiene kits. Households who lived close to admitted cholera cases but without admissions in their own households were anecdotally dissatisfied that they did not receive kits which could have led to discontent or stigmatisation of households with cholera cases.

### Domain 3: context

**Fig. 4:**
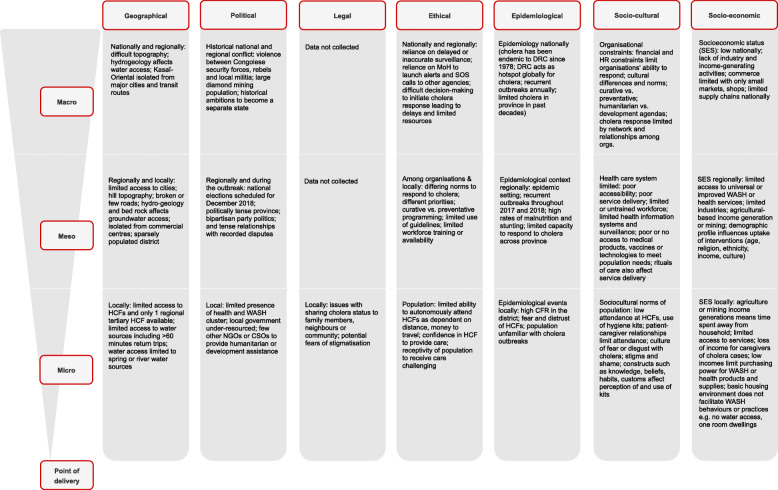
Contextual influences and events that may have influenced the implementation and participant response to hygiene kit distribution during a cholera outbreak in Kasansa, Kasaï-Oriental, DRC

Contextual events and influences unique to and across the macro, meso and micro levels were extracted from SSIs and intervention reports, conceptualised in Fig. 4, and supplement reported findings among the other domains and dimensions. Key examples of contextual factors affecting the implementation of and participant response to the intervention included: the limited surveillance leading to delayed response initiated after case confirmation **[Barrier 1]**, the geographical context of limited access to WASH infrastructure and distances to water sources **[Barrier 16]**; the socioeconomic status of the population, which affected purchasing power and explains a preference for help with food and clothing in addition to hygiene kits **[Barrier 14]**; and the local political context which affected the limited resources available for cholera response programmes and lack of other actors available to respond.

## Discussion

This process evaluation of hygiene kit distribution during a cholera outbreak identified numerous barriers to effective implementation of and participant response to the intervention, and to our knowledge is the first published process evaluation of a cholera outbreak response by MSF. During the late-2018 cholera outbreak in Kasansa, Kasaï-Oriental, DRC, it was observed that only 52% of admitted suspected cholera cases received a hygiene kit intervention. Although the majority of admitted patients in receipt of a hygiene kit had received the intervention on the day of admission, the delay of the overall response, delayed supply of hygiene kits and insufficient quantities of hygiene kits available limited the intervention’s coverage and utility, and may have diminished the effectiveness of the intervention. Overall, our process evaluation demonstrated that a large proportion of households either did not receive the kit or received the kit after the incubation period, or at least during the symptomatic period of the first case in the household, meaning that much of the infectiousness window from primary to secondary generation cholera cases within the household was not mitigated, nor would there be a reduction in the incidence of cholera among the population globally, as depicted in the ToC.

The analysis identified four key points in the ToC where barriers affected the implementation of and response to the hygiene kit intervention, and the ultimate reduction in cholera incidence and associated cholera morbidity and mortality (Fig. 2). In our case, the relative effect on within-household transmission will be reportedly separately in a parallel cohort study.

### Barriers to hygiene kit arrival at the project site

The MSF response was delayed and launched 16 weeks into the outbreak, clearly diminishing any potential effect of a cholera outbreak response. Capacity to deliver the hygiene kits to the intervention site was largely lacking at the national level within MSF, and although hygiene kits are part of the emergency preparedness stocks in the capital city Kinshasa, insufficient quantities were delivered. This highlights the need for expansion of emergency preparedness supplies at the national level or regionally at other MSF project sites (e.g. in Kananga [77]) with a reliable supply chain and transport of standard components required in cholera responses [36, 78], as well as the need for market supply chain analysis in key sites nationally to increase the ability for local purchasing and supply [79].

### Barriers to hygiene kit distribution at the HCFs

The distribution of hygiene kits was intended to target those households most at risk, but insufficient supplies and the decision to only distribute from CTUs and not the ORPs limited coverage and availability of the intervention to this population at risk. ORPs are typically located remotely and only provide rehydration to cases [30], however, they also may include 1–2 beds for case management and households may return home from these HCFs without attending the more centralised CTU or HCF. The exclusion of ORPs limited the intervention reach, and there is an opportunity to distribute hygiene kits to case-households from these HCFs and, in future responses, ORPs may need to be included for a more case-centred approach.

MSF guidelines specify that timely distribution is at admission [30]: CHW confusion on the timing of distribution had an effect on when kits were distributed, suggesting a need to reinforce the recommended delivery times in future trainings. The design of the hygiene kit demonstrations and health promotion should also be reflected upon to ensure the timely and sustained use of the hygiene kits in patient’s homes, and overall WASH intervention uptake. Evidence has shown that more participatory and engaging approaches, instead of simple health messaging [80, 81], are also needed to motivate and increase households’ ability to mount disease control efforts [69] and future programmes should adopt such frameworks.

### Barriers transferring hygiene kits to patient households

In many cases, admission of suspected cases to HCFs came 0–3 days after the onset of symptoms, but timely presentation of cases at HCFs remains a major issue experienced in cholera outbreaks [82]. Interviewees reported that many cases arrived without accompanying household members and thus were impeded from taking kits directly home whilst they were admitted. The time delay from receiving the kit, distance of HCFs to households and burden of transferring the kit home (i.e. bulky to transport for long distances) limited prompt use. These factors in turn may diminish the ability of the hygiene kits to reduce transmission within the transmission window from cases to household contacts.

One potential solution could be to increase active case finding at the district level and employ more CHWs to encourage cases to attend and household members to accompany suspected cases to HCFs. Another option could be to deploy rapid response teams (RRTs) to directly deliver hygiene kits and other interventions (e.g. other WASH materials, oral cholera vaccination (OCV) or antibiotic prophylaxis) to case households and the surrounding population at risk [27–29, 78], especially in densely populated or urban settings [28]. However, expanding the coverage of an intervention beyond patient-centred delivery may not be feasible for some organisations and a wider case-area targeted intervention (CATI) approach may require intensive case identification, a highly mobile response and high financial resources.

### Barriers to hygiene kit use by households

Recall of POU water treatment demonstrations was limited, and this may have curtailed the use or adherence to use of the Aquatabs™ chlorine tablets or P&G Purifier of Water™ flocculant disinfectant, as observed in other studies [44, 83]. Additionally, there was a limited supply of consumables in the kits and larger households found it difficult to maintain use for longer than a few weeks. Cholera contamination in the household can be sustained whilst cases are shedding bacteria for up to 14 days after onset of symptoms [20], and maintained use of interventions is required to reduce transmission [84]. This questions the use of standardised kits for variable household sizes, and suggests the additional need for contextual adaptations to be made to WASH intervention design considering household sizes, preferences and cultural norms [85].

Lastly, the hygiene kit intervention was designed to target key within-household transmission routes by treating contaminating drinking water and enabling improved hygiene practices. However, the intervention relies on a reliable supply of water and in sufficient volumes to facilitate hygiene practices [86]. Water sources were limited amongst this population, with distances >5 km return trip for most households, and volumes of water available for drinking, cooking and hygiene were broadly low, as seen in much of DRC [87]. Hygiene kit distribution is only one part of response efforts to reduce transmission and incidence of disease. Inadequate or limited access to WASH infrastructure will affect cholera prevention and control efforts [6], and may have interacted with the effect of the hygiene kit intervention, while other potentially important transmission routes have been ignored. Environment-to-human transmission of cholera directly from contaminated water sources [88], contact with faeces in the environment or lack of sanitation [18], fly transmission [89, 90] and safe burial practices [91], were not targeted with the hygiene kit intervention.

### Successes of hygiene kit distribution

Although several barriers limited the effectiveness of the overall intervention, it was noted that the intervention was well received by households that did receive the hygiene kit, and interviewed households were observed to and self-reported that demonstrations at the CTUs were clear and easy to understand. All components of the kit were used but in varying frequencies. Soap, jerrycans and the handwashing device were reportedly and observably used the most, and high adherence to using the handwashing device was reported in SSIs. Overall, hygiene kit distribution successfully translated to reported and observed improvements in household WASH knowledge and practices, as expected in the Outputs and Outcomes listed in the ToC.

Existing research has provided examples where both the kit and its components can reduce the incidence of cholera [24, 47–50, 92, 93]. With one study in Bangladesh finding cholera-specific hygiene promotion and hygiene kit distribution - a similar case-centred strategy to this MSF response- among admitted cases and households contacts leading to a 50% reduction in cholera incidence among household contacts of cases [25]. Although barriers to coverage and utility have been identified in this study, the hygiene kit may be an effective rapid and short-term measure in contexts such as DRC, particularly where longer-term WASH improvements are under-resourced [8], and targeting interventions to case households where risk of transmission is higher may thus be more efficient [12–14, 94].

### Limitations

The distribution of hygiene kits was implemented as a programme, not a research study, and accordingly this left many factors of intervention delivery open to change and interpretation by the implementers, rather than under control of the study staff. Although this allowed for the process evaluation to reflect real-world conditions, it limits the ability to rigorously test and draw certain conclusions on what could be effective models for delivery and adoption of the intervention by the population. Evidence around the effectiveness of WASH interventions in cholera prevention and control is limited [24], particularly from emergency contexts [95], and although the results here show the distribution of kits is a feasible response in this setting, a true assessment of this intervention to reduce cholera transmission would require a more rigorous study design.

Another limitation of the study is the reflexivity and bias of the researchers. It is possible that as the researchers were MSF staff and working closely under intense conditions with the intervention team, the relationship between the enumerator and interviewees may have exacerbated bias in their interactions and/or their reporting of events. There are already numerous challenges noted from conducting research in humanitarian contexts [95, 96], but the close relationship of the evaluation team to the implementers and pressures of the context may have resulted in social desirability bias on the side of the interviewees and interviewer bias in the side of enumerators. Most data were self-reported, and saturation of the data was reached quickly in our study population, potentially due to reporter and social desirability bias. Households who were interviewed may also have been more likely to recall positive experiences to the MSF-related, and thus intervention-related, evaluation team, leading to an inflation of responses. It has also been argued that interviews and data collected on personal behaviours such as handwashing are often overestimated [45, 92].

Additionally, although a snapshot of the contextual events and influences have been captured during our study, it is difficult to both capture all events and influences that may impede or strengthen the effect of an intervention [93]. Our approach assumed a relatively stable relationship between context, implementation and participant response [97] and have taken a snapshot of one point in time. However, some of the identified factors may have existed prior to the intervention, or there may have been a dynamic relationship that emerged during implementation which does not capture the effect of the context on the programme [50, 98]. The causal pathway of the relationship between the three domains of context, implementation and participant response may not be fully understood and this study design may not have provided the means to understand the process. Despite these limitations, the results of the study are informative, and we have triangulated across multiple data collection methods.

## Conclusions

Hygiene kit distribution is a promising intervention for cholera control. The integration of a WASH intervention at the point of admission of suspected cases is new in cholera control efforts, particularly in outbreaks and complex emergencies. This study has shown that it is possible to distribute interventions from the HCF and employ case-centred WASH interventions. However, the programme we evaluated suffered from barriers to the timely supply, inadequate availability and consequent coverage of the hygiene kits. These issues warrant consideration in subsequent cholera responses, the development of new guidelines, training of new staff and integration of these findings in national and organisation-specific cholera control efforts. Further research is also required to identify ways to improve implementation and delivery of this promising intervention.

## Data Availability

The datasets generated and/or analysed during the current study are available from the corresponding author on reasonable request.

## Abbreviations

AR: Attack Rate
CFR: Case Fatality Rate
CHW: Community Health Worker
CTC: Cholera Treatment Centre
CTU: Cholera Treatment Unit
DRC: Democratic Republic of Congo
HCF: Health Care Facility
MSF: Médecins Sans Frontières
NGO: Non-Governmental Organisation
OCV: Oral Cholera Vaccination
ORP: Oral Rehydration Point
PNECHOL-MD: Programme National d’Elimination du Choléra et de Lutte contre les autres Maladies Diarrhéiques
POU: Point of Use
RRT: Rapid Response Teams
SSI: Semi-Structured Interview
ToC: Theory of Change
WASH: Water, Sanitation and Hygiene
W: Epidemiological Week
